# Microcirculatory Changes in Experimental Models of Stroke and CNS-Injury Induced Immunodepression

**DOI:** 10.3390/ijms20205184

**Published:** 2019-10-19

**Authors:** Sarah Lunardi Baccetto, Christian Lehmann

**Affiliations:** 1 Department of Anesthesia, Pain Management and Perioperative Medicine, Dalhousie University, Halifax, NS B3H 4R2, Canada; sarah.baccetto@usp.br; 2 Department of Microbiology and Immunology, Dalhousie University, Halifax, NS B3H 4R2, Canada; 3 Department of Physiology and Biophysics, Dalhousie University, Halifax, NS B3H 4R2, Canada; 4 Department of Pharmacology, Dalhousie University, Halifax, NS B3H 4R2, Canada

**Keywords:** stroke, microcirculation, inflammation, immunodepression

## Abstract

Stroke is the second-leading cause of death globally and the leading cause of disability in adults. Medical complications after stroke, especially infections such as pneumonia, are the leading cause of death in stroke survivors. Systemic immunodepression is considered to contribute to increased susceptibility to infections after stroke. Different experimental models have contributed significantly to the current knowledge of stroke pathophysiology and its consequences. Each model causes different changes in the cerebral microcirculation and local inflammatory responses after ischemia. The vast majority of studies which focused on the peripheral immune response to stroke employed the middle cerebral artery occlusion method. We review various experimental stroke models with regard to microcirculatory changes and discuss the impact on local and peripheral immune response for studies of CNS-injury (central nervous system injury) induced immunodepression.

## 1. Introduction

Stroke is the second-leading cause of death globally [1], accounting for approximately 10% of all deaths worldwide [2]. In the United States, every 40 s a patient suffers from stroke, and one patient dies from stroke approximately every 4 min [3]. Although there has been a decrease in stroke incidence and mortality over the past decades [4], the overall burden of stroke remains high.

There are two main types of stroke: ischemic and hemorrhagic, with approximately 85% of the total number of prevalent strokes being ischemic [5]. Between 1990 and 2016, there was a decrease in the age-standardized death rates from stroke (36.2%) [5], but the estimated global lifetime risk of stroke had a relative increase of 8.9%, with the risk of ischemic stroke being greater than that of hemorrhagic stroke [6].

A systematic analysis of the burden of neurological disorders from 1990 to 2015 found that stroke was responsible for 47.3% of total DALYs (disability-adjusted life-years) and the majority of deaths (67.3%) [7]. Projections indicate that by 2030 stroke will be responsible for almost 12 million deaths, 70 million survivors, and over 200 million DALYs lost globally each year [8].

The decline in stroke mortality observed in recent decades is due to an improvement in the prevention of stroke obtained through to an improvement in the management of risk factors [4], such as control of diabetes mellitus and hypertension and smoking cessation programs.

Stroke is also the leading cause of disability in adults [9]. A cohort study [10] determined the frequency of symptomatic complications up to 30 months after a stroke, and found that 85% of the patients experienced at least one complication while they were in the hospital. After hospital discharge, there was a high frequency of infections, falls, pain, and symptoms of depression and anxiety. Most complications developed in the first 6 weeks after the stroke.

Another study showed that there is a strong association between complications after a stroke and a poor outcome (severe disability and death), since medical complications were evidently a leading cause of death in patients who suffered from acute ischemic stroke [11]. They also suggested that these complications may prevent recovery. Specifically, infections acquired after a stroke, especially pneumonia, are the leading causes of death [12].

In that context, systemic immunodepression is considered to contribute to increased susceptibility to infections post-stroke. Prass et al. demonstrated in a mouse model of cerebral ischemia [13], that CNS-injury due to ischemia induced a rapid and long-lasting inhibition of cell-mediated immunity and immunodepression via the hypothalamic-pituitary-adrenal axis (HPA) and the sympathetic nervous system (SNS), resulting in lymphopenia and disturbed lymphocyte and monocyte function. Notably, 1–3 days after the stroke, the animals developed spontaneous bacterial infections (mostly pneumonia and sepsis). In the same study, it was shown that the inhibition of the SNS prevented infections and significantly reduced the high rate of mortality of the model. Studies also show strong indications of immunodepression following ischemic stroke in humans (CNS injury-induced immunodepression syndrome (CIDS)), providing evidence of an immediate suppression of cell-mediated immunity [14].

Taking that into consideration, an immunotherapeutic approach, such as immunomodulatory drugs, may be beneficial after a stroke, helping to prevent or reverse immunodepression and decreasing the risk of infections [13]. The review will contrast and compare different experimental stroke models used to study CIDS, focusing on four of the most common models used currently: middle cerebral artery occlusion (MCAO), photothrombotic stroke (PTS), endothelin-1 (ET-1), and hypoxia-ischemia (HI). For this review, we researched academic databases and search engines, PubMed and Google Scholar, for the keywords “stroke”, “ischemia”, “experimental models”, and “immunosuppression”.

## 2. Animal Models of Ischemic Stroke

### 2.1. Middle Cerebral Artery Occlusion Model

The middle cerebral artery occlusion model is the most frequently used experimental model of ischemic stroke [15,16]. This model was first introduced and described by Koizumi and colleagues in 1986 in rats [17], and since then it was modified frequently. Among the different models for occlusion of the MCA, intraluminal suture is the most common method used in rodents [18]. Longa et al. employed modifications concerning different types of filaments to occlude cerebral arteries (kind, coating, and length) and access route [19]. Later, this model was also adopted for mice [20] and increasingly applied there.

In this model a filament is inserted into the internal carotid artery (ICA) and advanced until it blocks the origin of the middle cerebral artery (MCA), interrupting the blood flow [16,19]. After the insertion, the filament can be left permanently in order to model permanent ischemia or, to model transient focal cerebral ischemia, it can be withdrawn after a certain period of time to induce reperfusion of the MCA [21,22]. This allows a precise control of the reperfusion and duration of ischemia [18].

Besides being less invasive and not requiring craniectomy, this model is also relatively easy to perform and provides infarctions that are reproducible [16]. It also mimics ischemic stroke in humans concerning its location, since most thromboembolic infarcts occur in the region of MCA in humans, and the penumbra it exhibits in the animal is similar to that of human stroke [18,23].

There are some disadvantages of the MCAO model that need to be taken into consideration: (1) it may result in incomplete MCAO due to inadequate filament size or length of insertion [22,24], and it may cause vessel rupture and subsequent subarachnoid hemorrhage (SAH) [16,24]; (2) it causes hypothalamic injury if MCAO lasts for 120 min or more, leading to hyperthermia which rarely occurs in humans [25,26]; and (3) there is a prompt reperfusion of the MCA after the removal of the filament, whereas in human ischemic stroke reperfusion of the vessel typically occurs gradually [27]. However, the prompt reperfusion observed in this model is similar to what occurs in endovascular thrombectomy [15], a therapy that has been showing favorable outcomes in recently published randomized controlled trials [28,29].

Additionally, there are other methods used for occlusion of the MCA. Embolic MCAO models closely mimic human stroke, since over 80% of stroke is caused by thrombosis or embolism [30]. Embolization is induced through the introduction of blood clots or artificial emboli most common to the origin of the MCA [30,31,32]. This model also allows the study of thrombolysis and thrombolytic therapies [31,32]. However, embolic MCAO is associated with higher variability in infarction size and location and higher mortality [30,31].

### 2.2. Photothrombotic Stroke Model

The photothrombotic stroke as a model of ischemic stroke was first introduced in rats in 1985 by Watson and colleagues [33], but since then it has been modified and applied in mice. The model is based on a photochemical reaction that induces thrombosis, leading to cerebral infarction. A photosensitive dye (e.g., Rose Bengal, erythrosin B) is administered intraperitoneally in mice or intravenously in rats followed by illumination of the brain with a light of a specific wavelength [33,34]. Photo activation of the dye leads to formation of oxygen free radicals, such as singlet oxygen and superoxide, resulting in endothelial damage and platelet activation and aggregation within the illuminated area [35]. This method causes a rapid progression of ischemic cell death in the irradiated area, with alterations of the cerebral blood flow (CBF) firstly in the areas of the infarct and then in remote sites [36].

It is possible to induce ischemia in a determined region of the brain with this model through the use of stereotactic coordinates [37]. There is no need for craniectomy since the light source can be applied directly on the skull of mice, characterizing PTS as a noninvasive, highly reproducible, and relatively easy model to perform with low animal mortality [18,38]. The management of the light intensity and duration also allows control over the degree of the ischemic lesion [39]. Additionally, this model is advantageous for cell characterization and functional studies since the infarction produced is of small size and has well-delimited boundaries [38].

However, there are some discrepancies when compared to acute human stroke. While the latter is characterized primarily by cytotoxic edema in the earliest stages of ischemia [40], the injury caused by PTS presents simultaneously acute cytotoxic and vasogenic edema due to microvascular injury and blood–brain barrier (BBB) breakdown [41]. Additionally, considering this model causes ischemic lesion with well-defined boundaries, it consequently generates little or no penumbra and collateral blood flow, which is typically present in human ischemic stroke. Since the penumbra is the main target for postischemia neuroprotective agents, PTS presents poor translational impact for the study of these agents [38].

Nonetheless, there has been modifications of the model that improve those aspects. Studies using a photothrombotic ring stroke model were able to produce a predefined area-at-risk surrounded by a ring of ischemic lesion, with pathomechanisms considered relevant to the evolution of clinical thromboembolic stroke with penumbra [42,43]. Another study using the PTS model was able to show a bordering zone around the infarcts by illuminating the brain with minimal light intensity [44]. Recently, Qian et al. performed a photothrombotic occlusion of the proximal middle cerebral artery in mice and were able to precisely visualize the penumbra not only surrounding, but also in, the lesions, which opens up the possibility of studying different therapeutic approaches with this model, such as thrombolysis and neuroprotectant agents [45].

Furthermore, different studies were able to induce PTS in conscious and freely moving rats [46] and mice [47], which allows analysis of various parameters post-stroke without the influence of anesthesia; more specifically, real-time CBF imaging and detection of motor deficits at different time points. Not only does this model makes it possible to evaluate neurobehavioral and physiological parameters throughout the induction of stroke but it also opens up the possibility of assessing the effects of early therapeutic interventions post-stroke [47].

### 2.3. Endothelin-1 Model

Another experimental model of stroke is based on the application of endothelin-1, a peptide that presents potent and long-lasting vasoconstrictive properties [48]. Several studies have shown that the application of ET-1 causes a rapid and significant decrease in CBF [49,50,51] that leads to ischemic lesion followed by gradual reperfusion [52,53], which closely resembles the CBF reduction and reperfusion that occurs in clinical stroke [53].

There are different methods that have been used for the application of ET-1: directly onto exposed MCA of the rat [49,52], through stereotactic injection into tissue adjacent to the MCA [53,54] or into the striatum or cortex [51,55], or onto the cortical surface of the brain [56]. This model allows the targeting of any brain region through the use of stereotactic injections, producing precise and reproducible ischemic focal ischemic lesions without disruption of the BBB [51]. It has been shown that the ischemic lesion provoked is dose-dependent [51,56], which allows some control over the extension of the damage. There is also evidence of a large penumbra area associated with ET-1-induced lesions [56].

The stereotaxic approach to inject ET-1, which has been the most used recently [22], requires a surgery that is less intrusive and causes minimal damage to facial or temporal muscles [54]. The animals do not exhibit postoperative eating disorders and recover their weight within a few days [50], and the technique is associated with minimal morbidity [53]. Furthermore, the guide cannulas can be implanted in advance, allowing ET-1 to be injected in conscious animals, thus making it possible to eliminate confounding effects of anesthesia and artificial ventilation in the development of the ischemic damage and the effects of potential therapeutic agents [57].

However, ET-1 receptors and ET-1 converting enzymes have been found not only in endothelial cells in the brain of rats, but also in neurons and astrocytes [58]. Additionally, findings suggest that ET-1 induces astrocytosis, and is involved in axonal degeneration after spinal cord injury [59,60], which may interfere with interpretation of neural repair experiments [61].

The majority of ET-1 stroke experiments have been done in rats. Although some studies have shown that ET-1 is significantly less potent in mice than rats, producing small infarcts and neurobehavioral deficits that were largely resolved within 3 days post-injury [55,62], there have been more recent studies that show efficacy in mice. Sozmen et al. were able to develop a viable subcortical white matter stroke model in mice mediated by ET-1 [63] and Roome and colleagues developed a reproducible model of focal ischemia in mice with measurable neurobehavioral deficits through ET-1 injections targeted to the forelimb motor cortex [64].

Furthermore, focal injection of ET-1 can be a useful model to study the mechanisms of cytotoxic edema induced by ischemic lesion and to investigate the potential to preserve axonal integrity of different compounds [51].

### 2.4. Hypoxia-Ischemia Model

Originally developed in the 1960s in adult rats, the Levine/Vannucci procedure of hypoxia-ischemia consists of ligation of the unilateral common carotid artery (CCA) followed by whole body hypoxia for a predetermined time [65]. In the 1980s, the method was modified and became popular as an experimental model of perinatal hypoxic-ischemic brain injury using rat pups [66]. It has been shown that neither the unilateral carotid occlusion nor the hypoxia alone are able to produce brain damage, but the combination of both leads to infarction [65,66].

This model does not require complicated surgical skills and it causes a significant reduction in CBF [67,68]. More specifically, Adhami and colleagues showed that combining hypoxia with unilateral occlusion of the CCA reduces the regional CBF to a degree similar to that of focal ischemia models and that it has persistent negative effects on cerebral reperfusion, but neither the carotid occlusion nor the hypoxia alone were able to significantly reduce CBF and it quickly returned to normal [67].

A common criticism of the HI model is concerning to its consistency, since there are reports of high variability in infarct volume [65,66,68,69]. However, recent studies suggest that controlling the body temperature or adjusting the duration of hypoxia produces more consistent brain injury in adult mice [70,71,72]. Additionally, Edwards et al. demonstrated that permanently occluding both the CCA and the external carotid artery (ECA) before hypoxia in P7 rat pups induces a more reproducible and larger infarct compared to CCA occlusion only [68]. There is also evidence that increasing the duration of hypoxia increases the degree of brain damage in P7 rat pups [73].

There is controversy regarding the effects of HI in adult versus young animals. Some studies suggest that the immature brains are less susceptible to HI injury when compared with mature brains [74] and that not only there is a progressive increase in frequency of brain injury with increasing age, but there is also a change in regional vulnerability to HI during development [75]. Other experiments, however, report that the brain damage produced by HI is more severe at either end of the age spectrum: Yager et al. demonstrated that the damage was more severe in 1- and 3-week old and 6 months old rats and less severe in juvenile animals (6- and 9-week old animals) [76], and Ikonomidou et al. showed that rats aged 4–14 days suffer the most injury while newborn rats and animals older than 20 days are less sensitive to HI injury [77]. Taking that into consideration, the duration of HI and treatment of brain injury need to be adjusted according to the developmental level of the animal [74].

Besides being a model that allows both the induction of infarct in adult animals and perinatal HI brain injury, it can be useful also for evaluation of long-term brain damage and neurological deficits [78], investigation of cellular and molecular mechanisms that occur after cerebral ischemia and possible therapeutic interventions [70], and for assessing therapeutic approaches to restore post-ischemic reperfusion [67].

## 3. Cerebral Microcirculation in Experimental Stroke

### 3.1. Middle Cerebral Artery Occlusion Model

**Figure 1:**
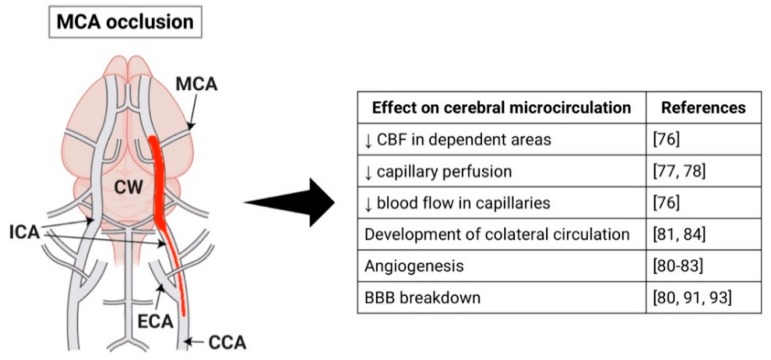
Schematic representation of the middle cerebral artery (MCAO) stroke model and its effects on the cerebral microcirculation. Figure created with Biorender (https://biorender.com).

Occlusion of the MCA results in reduction of CBF in the dependent areas [79]. Studies show a significant reduction in capillary perfusion in the ischemic region of the brain following MCAO [80,81]. However, other research groups have shown that most capillaries do not suffer reduction of perfusion after MCAO, but they present decreased blood flow [79] (Figure 1). Vogel et al. demonstrated that, during the first hours after MCAO, the plasma perfusion of most capillaries is maintained at a lower rate but later non perfused areas slowly replace the perfused areas [82].

Additionally, Lin and colleagues showed that MCAO has different effects on large and small vessels of rats: cerebral blood volume (CBV) increased in large vessels whereas in small vessels there was an initial decrease in CBV and vascular density and increase in vascular size and, at later time points, CBV and vascular density were increased in the outer layers of the infarcted area [83]. A recent study showed similar results and also reported two phases of CBV increase after MCAO: an early phase that depends on the development of collateral circulation and a late phase caused by angiogenesis [84]. Other studies have also observed signs of angiogenesis in the ipsilateral cortex after ischemia [83,85,86] (Figure 1).

Collateral circulation may also promote reperfusion of ischemic regions [87]. Armitage et al. demonstrated the presence of persistent collateral blood flow to the ischemic region provided by anastomotic connections between the ACA (anterior cerebral artery) and MCA, but these connections disappear after spontaneous reperfusion [88]. In another study, there was no recruitment of collateral channels in the ischemic core, but they were present immediately after MCAO in normal and penumbra areas and provided blood flow to the ischemic region [89]. The same group also identified three different types of collateral channels (CCs): persistent, impermanent, and transient, but only the persistent channels were able to maintain the CBF in its surrounding region, while regions supplied by impermanent and transient CCs presented gradually decreasing CBF [89].

Besides changes to brain hemodynamics after an ischemic insult, there is also a complex local inflammatory response. Initially, the ischemic injury causes activation of transcription factors that promote up-regulation of pro-inflammatory cytokines and chemokines which, in turn, stimulate the expression of adhesion cells and consequently infiltration of leukocytes [90]. Ritter et al. demonstrated that after MCAO, leukocytes accumulate in the brain and there is a significant increase in the number of rolling and adhering leukocytes associated with lower blood shear rates in the reperfused cerebral venules [91]. Other studies have also shown that leukocytes contribute to the extension of the brain injury after MCAO [92,93]. Moreover, activated leukocytes and the vascular endothelium are sources of reactive oxygen species (ROS) that act as signaling molecules and may further increase the injury [94,95].

Furthermore, there is evidence of BBB breakdown after MCAO, which may contribute to secondary damage [83,94,96] or be a potential therapeutic target. A disrupted BBB may cause leakage of inflammatory and antigenic products from the brain that can promote a systemic inflammatory response [90].

### 3.2. Photothrombotic Stroke Model

**Figure 2:**
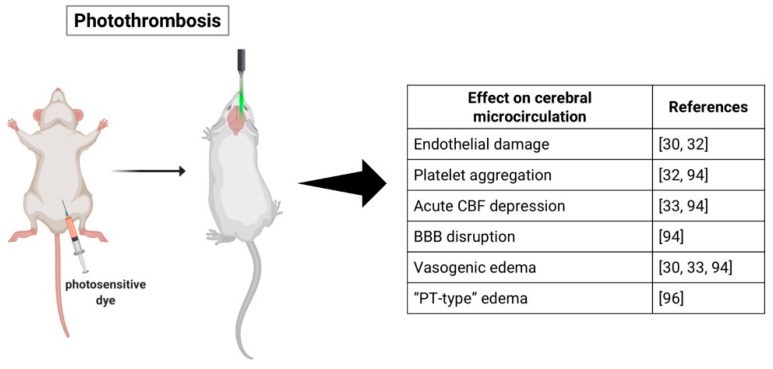
Schematic representation of the photothrombotic stroke (PTS) model and its effects on the cerebral microcirculation. Figure created with Biorender (https://biorender.com). Cerebral blood flow, CBF; blood–brain barrier, BBB.

Infarction induced by the PTS model appears to be a result of microvascular injury initiated by endothelial membrane damage [97]. The interaction between the light and the photoactive dye leads to formation of singlet oxygen molecules that induce peroxidative reactions, which are most likely the cause for endothelial membrane damage and may inactivate endothelial enzymes [33,35]. Consequently, platelets aggregate in both pial and parenchymal vessels causing an acute depression in CBF, primarily in the areas close to the infarct and later spreading to remote regions [35,36,97]. Subsequently, the BBB is disrupted, leading to increase in water content and formation of vasogenic edema, which may cause compression of the microcirculation and exacerbate the ischemic damage [33,36,97] (Figure 2).

Different studies, however, report that platelet aggregation and thrombi appear not to be essential for the development of the ischemic injury [37,98]. Frederix and colleagues suggest that the infarct might develop due to BBB damage resulting in parenchyma injury instead of microvascular thrombosis [98]. Conversely, Yushmanov et al. inferred blood cannot reach the ischemic core due to complete microvascular thrombosis, differentiating the “PT-type” edema occurring in the PTS model from a “pure” vasogenic edema seen in MCAO [99]. Additionally, another group found that in response to the ischemic injury provoked by photothrombosis, the brain’s microvasculature altered its orientation from the outer regions into the infarct area, with vessels that angled towards the center of the infarct [100].

Lymphocytes infiltrate the ischemic lesion first in the border zone and later in the infarct center, followed by macrophage invasion [101]. Schroeter et al. demonstrated that the initial phagocytic response in the area surrounding the infarct is mainly derived from microglia [102]. A study comparing the local inflammatory response in MCAO and PTS showed that photothrombosis induced a delayed microglial and astroglial activation and delayed accumulation of activated microglia in the ischemic core, but the PTS model caused an increased inflammatory response, demonstrated by higher levels of cytokines and chemokines and increased infiltration of circulating leukocytes [103].

It has been shown that brain inflammation following photothrombosis persists for up to 14 days, during which there is lymphocyte infiltration, higher levels of ROS, and up-regulation of proinflammatory cytokines [104]. Furthermore, Feng et al. showed that the profile of cells infiltrating the brain of humans that died within 7–14 days after stroke onset correlates to the lymphocyte infiltration in the PTS model, indicating that inflammatory infiltration persists during the late stages of ischemia in both stroke patients and mice models [104]. There is also evidence that both resident (microglia) and peripheral immune cells accumulate in secondary sites of neurodegeneration over 14 days post-stroke [105].

### 3.3. Endothelin-1 Model

**Figure 3:**
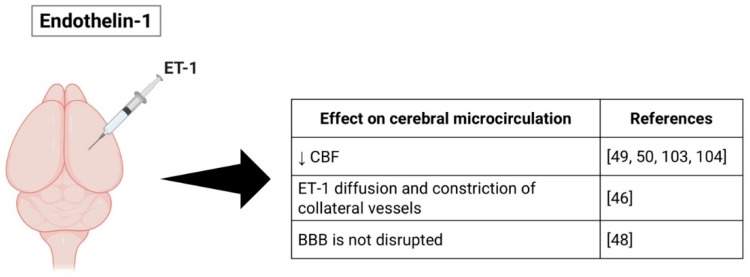
Schematic representation of the endothelin-1 (ET-1) stroke model and its effects on the cerebral microcirculation. Figure created with Biorender (https://biorender.com).

Application of exogenous ET-1 is capable of rapidly reducing blood flow to pathologically low levels and its effects are long lasting, since the reduction in CBF is maintained during long periods [52]. Studies show that cortical CBF remained depressed for 16 h, returning to contralateral levels after 22 h [53] and also that the decrease in ipsilateral CBF was evident at 48 h after ET-1 injection [106]. Other reports, however, demonstrated a faster recovery of CBF [107]. Robinson et al. suggests that ET-1 diffuses and constricts collateral vessels, reducing the compensatory effect of collateral blood flow [49]. Application of ET-1 has also been shown to not cause disruption of the BBB [51] (Figure 3).

A study from 2003 demonstrated that ET-1-induced ischemia caused neuronal death 6 h after ET-1 injection but no neutrophil recruitment, and a delayed activation and recruitment of microglia and macrophages at 72 h [51]. On the other hand, Weston and colleagues showed in an unanesthetized ET-1 ischemia model, an increase in neutrophil infiltration over time, peaking at day 3, a positive correlation between the infarct volume and neutrophil infiltration and the occurrence of phagocytosis of neutrophils by macrophages as early as one day after ischemia and increasing with time [108]. Furthermore, other authors reported infiltration of lymphocytes 14 days post-stroke [104].

### 3.4. Hypoxia-Ischemia Model

**Figure 4:**
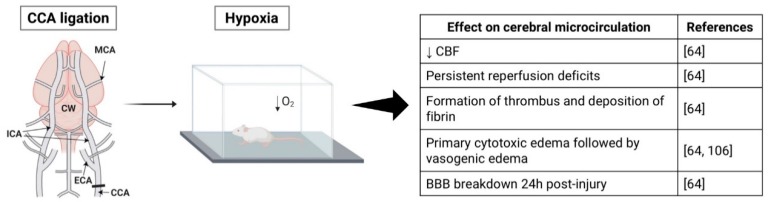
Schematic representation of the hypoxia-ischemia (HI) stroke model and its effects on the cerebral microcirculation. Figure created with Biorender (https://biorender.com).

Cerebral ischemia combined with hypoxia leads to the reduction of CBF and persistent reperfusion deficits [67]. Adhami et al. reported that HI can induce the formation of thrombi in the microvasculature and deposition of fibrin within the brain [67]. CNS injury induced by HI has also been shown to promote a biphasic evolution of edema, a primary cytotoxic edema followed by vasogenic edema [67,109] (Figure 4).

Studies suggest that cytotoxic edema occurs in two phases: the first one due to suppression of cell metabolism (neurons and glial cells) and neuronal swelling and a second phase provoked by activated glial cells [109,110]. Furthermore, since cytotoxic edema has been shown to be accompanied by a preserved BBB, vasogenic edema was attributed to a breakdown of BBB 24 h after CNS injury [67]. Another explanation is that necrotic and apoptotic cells gradually enhanced the vasogenic edema [109].

Following the initial CNS injury, the damaged areas are occupied by both activated resident microglia and peripheral macrophages, which have a negative effect on neuronal survival [72]. Additionally, studies suggest that HI induces local production of proinflammatory cytokines, such as IL-6 and IL-1β [67], and leads to a chronic state of inflammation, evidenced by persistence of microglia/macrophages, lymphocytes, and astroglia activation for up to 35 days [111].

## 4. Peripheral Immune Response

Following the initial local inflammatory response to cerebral ischemia, peripheral inflammatory processes and immune responses can contribute to stroke outcome. Specifically, there is growing evidence of an immunosuppressive state following stroke since CNS injury disturbs the balance between the immune system and CNS [112]. In humans, different studies present markers of CIDS: lymphopenia, impaired T- and NK-cell activity, functional deactivation of monocytes, and atrophy of the spleen [14,112,113,114]. Moreover, the size and severity of stroke has been correlated with immunosuppression magnitude [14,114].

Several studies have also demonstrated the establishment of immunodepression in animals following experimental stroke (Table 1). Prass and colleagues demonstrated for the first time that cerebral ischemia induces a long-lasting depression of cell-mediated immunity and that results in spontaneous bacterial infection [13]. More specifically, they suggest that a reduced production of IFN-γ by impaired NK and T cells is responsible for the stroke-induced deficiency in antibacterial defense [13]. Offner et al. also reported a stroke-induced immunosuppression manifested within days due to a reduction in activation of T cells and loss of T and B cells in the spleen and thymus [115].

Additionally, Liu et al. investigated the profile of NK cells in the brain and periphery following MCAO in mice, and reported an increase in NK cells in the brain that contracted afterward, a rapid decline in NK cells in the spleen, and spleen atrophy in the acute phase of stroke, indicating differences between NK cells in the brain and periphery [113]. The same study also showed that blocking adrenergic and HPA axis innervation of NK cells in the periphery enhanced the immune defense mediated by NK cells [113].

Furthermore, findings suggest that post-stroke immunodeficiency occurs primarily due to an activation of the SNS. Pharmacological inhibition of the SNS enhanced cellular immune responses following MCAO, i.e., it prevented lymphocyte apoptosis, lymphopenia, and monocyte deactivation, and prevented bacterial infections [13]. Offner and colleagues also speculate that the damaged brain triggers the initial inflammatory response through sympathetic neural stimulation, ultimately exhausting the immune defenses, leading to immunosuppression [90].

However, the vast majority of studies focused on the peripheral immune response to stroke employed the MCAO method of inducing cerebral ischemia. Our group has demonstrated that the endothelin-1 and HI models also induced CIDS. HI injury induced a reduction in the number of adhering leukocytes in intestinal microcirculation and a decrease in levels of proinflammatory cytokines (TNF-α and IL-6) [116]. Similarly, CNS injury provoked either by ET-1 or HI significantly impaired leukocyte-endothelial interactions in intestinal microcirculation, and brain injury followed by induced endotoxemia caused a major reduction in leukocyte–endothelial interactions [117]. Ultimately, an increase in infarct size correlated with stronger immune suppression [117].

## 5. Discussion

Stroke is a complex and heterogenous disorder in humans, often associated with other comorbidities and with high variability. In that context, experimental stroke models need to take several questions into consideration, and no animal model can cover all variables inherent to human stroke. In fact, the vast majority of research for neuroprotective therapies that were successful in animal models failed to translate in clinical trials [118,119]. Nevertheless, research with animal models has contributed significantly to the current knowledge of cerebral ischemia and its effects, and continues to be an indispensable tool to study stroke physiopathology and novel therapies [18,120]. Moreover, there are some computational and mathematical models that help understand stroke pathophysiology. Goodall et al. used a computational model of the primary sensorimotor cortex to induce acute focal lesions and examine perilesion excitability and cortical map reorganization immediately after the lesion and over long term [121]. Another group built a mathematical model to study the main mechanisms involved in the development of cytotoxic edema, more specifically the influence of ionic current on cell swelling during stroke [122].

Nonetheless, animal models of stroke present many characteristics that are common in human stroke, such as the concept of evolving damage after the ischemic injury, even though different species and strains of animals can affect the experimental outcome [32,61]. Overall, the most common broad subtype of stroke in humans is caused by occlusion of the MCA [23,32], thus the MCAO model is considered to be clinically relevant and is the most commonly used model. However, the surgery requires experience, and the results are substantially variable regarding infarct volume and behavioral deficits within and between mice strains [61]. Furthermore, Howells et al. pointed out that MCAO can easily induce large infarcts in various brain structures, modeling more closely malignant infarction than human stroke, which are usually smaller and associated with some recovery [32].

Another model that has been slowly replacing MCAO is the photothrombotic stroke [103]. Photothrombosis induces infarcts that are smaller and consistent and do not affect deep structures, which is more similar to what happens in humans, besides requiring minimal surgical manipulation [61,103,123]. This model also allows stereotactic placement of the infarct in determined areas [61]. However, there are also disadvantages with this model, such as occurrence of microvascular injury, BBB breakdown, and simultaneous vasogenic and cytotoxic edema [32,61,123]. The endothelin-1 model also allows stereotactic placement of the infarct in a determined region, but there is limited control of the intensity and duration of ischemia [32].

The impact of the surgery and anesthesia also need to be taken into consideration. Anesthesia is required at some point in all models of stroke whereas human stroke patients are not usually anesthetized [32]. Furthermore, it has been shown that anesthesia has neuroprotective and preconditioning effects [32]. Additionally, the surgery itself may induce inflammatory responses that can confound results. Therefore, models that enable induction of stroke without anesthesia, such as PTS and ET-1, might be more relevant.

Although the vast majority of findings regarding the peripheral immune response to experimental stroke was based on the MCAO model, we suggest that stroke-induced immunosuppression can be studied with any experimental stroke model. Liesz et al. demonstrated that the size of the infarct is the primary determinant of systemic immune alterations following ischemia, and that neither location of the injury or model have a major impact [124]. Hence, any model that produces an infarct of significant size can be used to study the peripheral immune response, and researchers should focus on the reproducibility of the model regarding location and size of the infarct, and minimal invasiveness.

## 6. Conclusions

It is widely acknowledged that no animal model mimics human stroke perfectly and each one reproduces different aspects of it, so researchers should choose what animal model is best and what outcome measures should be used based on the aim of the research. In summary, the lack of translation between the animal work and clinical benefits does not lie in the animal models, but in how we use the models and how we apply this knowledge to the design of clinical trials [125].

## Figures and Tables

**Table 1:** Summary of peripheral immune responses to different experimental stroke models.

Model	Findings	References
MCAO	Long-lasting depression of cell-mediated immunity resulting in spontaneous bacterial infection	[13]
	Reduced production of IFN-γ by impaired NK and T cells	[13]
	Reduction in activation of T cells and loss of T and B cells in the spleen and thymus	[115]
	Rapid decline in NK cells in the spleen and spleen atrophy in acute phase of stroke	[113]
	Blockage of adrenergic and HPA axis innervation of NK cells in the periphery enhanced immune defense mediated by NK cells	[113]
	Pharmacological inhibition of the sympathetic nervous system (SNS) enhanced cellular immune responses following MCAO	[13]
HI	Reduction in the number of adhering leukocytes in intestinal microcirculation	[116]
	Decrease in levels of pro-inflammatory cytokines	[116]
HI and ET-1	Impairment of leukocyte–endothelial interactions in intestinal microcirculation	[117]
	Increase in infarct size correlated with a weaker immune response	[117]

## Abbreviations

CNS	Central nervous system
DALYs	Disability-adjusted life-years
HPA	Hypothalamic-pituitary-adrenal
SNS	Sympathetic nervous system
CIDS	CNS injury-induced immunodepression syndrome
MCAO	Middle cerebral artery occlusion
ICA	Internal carotid artery
MCA	Middle cerebral artery
SAH	Subarachnoid hemorrhage
PTS	Photothrombotic stroke
CBF	Cerebral blood flow
BBB	Blood-brain barrier
ET-1	Endothelin-1
HI	Hypoxia-ischemia
CCA	Common carotid artery
ECA	External carotid artery
CBV	Cerebral blood volume
ACA	Anterior cerebral artery
CCs	Collateral channels
ROS	Reactive oxygen species
IL-6	Interleukin-6
IL-1β	Interleukin-1β
NK	Natural killer
IFN-γ	Interferon-γ
TNF-α	Tumor necrosis factor alpha
